# The Italian Version of the New General Self-Efficacy Scale (NGSES): Structural Validity, Psychometric Properties, and Measurement Invariance

**DOI:** 10.3390/jcm14061988

**Published:** 2025-03-14

**Authors:** Alessandro Alberto Rossi, Stefania Mannarini, Federica Taccini, Gianluca Castelnuovo, Giada Pietrabissa

**Affiliations:** 1 Department of Philosophy, Sociology, Education, and Applied Psychology, Section of Applied Psychology, University of Padova, 35131 Padova, Italy; a.rossi@unipd.it (A.A.R.); stefania.mannarini@unipd.it (S.M.); federica.taccini@unipd.it (F.T.); 2 Center for Intervention and Research on Family Studies (CIRF), Department of Philosophy, Sociology, Education, and Applied Psychology, Section of Applied Psychology, University of Padova, 35131 Padova, Italy; 3 Department of Psychology, Catholic University of Milan, 20123 Milan, Italy; gianluca.castelnuovo@auxologico.it; 4 Clinical Psychology Research Laboratory, IRCCS Istituto Auxologico Italiano, 20145 Milan, Italy

**Keywords:** self-efficacy, confirmatory factor analysis, measurement invariance, clinical psychology

## Abstract

**Background/Objectives:** General Self-Efficacy (GSE) refers to an individual’s belief in their overall ability to perform effectively across various situations. Research shows that GSE is a key predictor of multiple outcomes, including psychological resilience, better health, and improved overall well-being across different populations and environments. Numerous tools have been developed to measure GSE, enhancing our understanding of self-efficacy and its broader implications. This study aimed to evaluate the psychometric properties of the Italian version of the New General Self-Efficacy Scale (NGSES) in a community sample of adults recruited through social media platforms. **Methods**: The NGSES was adapted into Italian following the back-translation procedure. A total of 811 participants (*mean* = 43.18; 68.8% females) completed the scale. A confirmatory factor analysis (CFA) was used to examine the factorial structure of the questionnaire. Adjusted item-total correlations and internal consistency were assessed using McDonald’s omega. Additionally, multi-group CFA was used to test factorial invariance across gender. **Results**: The trimmed model exhibited a strong fit to the data: RMSEA = 0.068, CFI = 0.994, SRMR = 0.043. An omega coefficient of 0.872 confirmed the scale’s strong reliability. Convergent validity was established by a moderate and significant correlation with the NGSES. Configural, metric, scalar, and latent means invariance across genders were all confirmed. **Conclusions**: The NGSES was found to be a valid and reliable tool, suitable for both clinical and research applications in the Italian context. It can also inform the development of educational and therapeutic interventions aimed at enhancing GSE in the general population.

## 1. Introduction

When considering the elements that influence human behavior, Bandura (1977) defined self-efficacy as an individual’s ability to exert control over events and do things in such a way as to be satisfied with the outcome [1]. The author proposed that perceived self-efficacy significantly influences people’s thoughts, behaviors, and emotional experiences, affecting both the initiation and persistence of coping behaviors.

Perceived self-efficacy can be conceptualized as either a trait-like or a state-like construct. The trait-like dimension pertains to beliefs about one’s capability to perform effectively across diverse situations, referred to as general self-efficacy (GSE). In contrast, the state-like dimension focuses on beliefs regarding the ability to succeed in specific tasks or contexts, known as specific self-efficacy or task self-efficacy (SSE). Both GSE and SSE are grounded in the same four informational sources: prior achievements, observational learning through others’ experiences, verbal encouragement or feedback from others, and physiological or emotional states [2,3,4].

The concept of generalized self-efficacy (GSE) was introduced by Sherer et al. (1982), who described it as shaped by an individual’s past experiences of success and failure [5]. According to Shelton (1990), individuals with high GSE tend to adopt a mastery-oriented approach to challenges, attributing successes to their efforts while being less likely to internalize blame for failures [6]. Conversely, individuals with low GSE often attribute failures to themselves and seldom take credit for their successes, fostering a sense of helplessness when facing a wide range of situations [5,7].

Enhancing GSE is vital for fostering psychological resilience and self-esteem, improving health, and enhancing overall well-being in everyday life [8,9,10,11,12,13]. Higher self-efficacy correlates with reduced stress, improved coping mechanisms, and greater success in navigating life challenges, such as professional transitions, academic demands, or parenthood [14,15]. Research also indicates that GSE is a significant predictor of various psychological outcomes, including levels of anxiety and depression [16].

In addition, individuals with high GSE are also more likely to set ambitious goals, persevere in achieving them, and engage in healthy behaviors, including regular exercise, balanced nutrition, and adherence to medical advice [17,18]. In contrast to this, those with low GSE perceptions are characterized by traits that undermine performance [19,20].

### Measures of General Self-Efficacy

The reliability and factor validity of the NGSES is supported by comparative studies [21], which demonstrated the superior psychometric properties of this scale compared to other tools, including the Personal Efficacy Scale [22], the Generalized Self-Efficacy Scale [7], and the General Perceived Self-Efficacy Scale [23].

Specifically, the NGSES has shown high internal reliability, with McDonald’s ω values exceeding 0.87 and Cronbach’s α typically ranging between 0.85 and 0.90, confirming its internal consistency and psychometric robustness.

In addition, the NGSES has demonstrated predictive validity by correlating with work engagement [24] and depressive levels in data collected from older adults with low vision [25]. It also aligns with constructs like self-judgment and self-response [26], representing a reliable and efficient tool for assessing GSE.

GSE has been explored in various fields, including organizational research [2,27], health [28,29], and education [30,31,32,33]. Despite this extensive focus, there remains a critical need for further studies employing robust and reliable methodologies to enhance our understanding of self-efficacy and its implications in community samples.

Researching GSE at a population level is essential for identifying groups that may be vulnerable to the negative consequences of low GSE, such as reduced mental health, poor coping strategies, and diminished motivation. By analyzing patterns across demographics (e.g., gender), researchers can uncover disparities in GSE and understand how these relate to specific challenges or contexts, such as unemployment, academic underachievement, or chronic illness. Ultimately, such research would aid in the development of evidence-based strategies to bolster resilience, improve well-being, and enhance life outcomes across various contexts.

At present, the NGSES has not been adapted for measuring self-efficacy among Italian individuals. Translating the NGSES from English into Italian and assessing its psychometric properties within this new cultural context would address this gap and provide a more recent, concise, and psychometrically robust alternative for assessing general self-efficacy in both research and clinical settings.

To this purpose, the present study aims to evaluate the structural validity and psychometric properties of the NGSES within a community sample of Italian adults. Furthermore, it investigates the measurement invariance of the NGSES across genders to determine whether the scale’s factor structure is consistently interpreted and functions equivalently for both males and females.

## 2. Materials and Methods

### 2.1. Translation and Cultural Adaptation

The translation and cultural adaptation process followed the guidelines for cross-cultural adaptation of self-report instruments that involved a series of steps [34,35]. (1) Translation: two independent Italian translators, without prior knowledge of the questionnaire’s theoretical framework, translated the NGSES from English into Italian. (2) Translation Synthesis: translated versions were compared and merged through discussions between the translators, ensuring consensus and maintaining theoretical equivalence with the original questionnaire. (3) Back-Translation: a professional back-translator, who was unaware of the original English version, translated the final Italian version back into English. This step allowed for a comparison between the back-translated and original versions to detect any inconsistencies or errors. (4) Expert Committee Evaluation: a panel consisting of all translators, two experienced health professionals (authors G.C. and G.P.), and a questionnaire validation expert (author A.A.R.) reviewed the translated and back-translated versions. This collaborative evaluation led to the development of a pre-final version. (5) Pre-Final Testing: the pre-final version was tested on a sample of 10 individuals from the general population to assess item clarity. Since no significant issues emerged, the questionnaire remained unchanged. The final version is available in the Supplementary Materials (Supplementary File S1).

### 2.2. Sample Size Determination

The sample size was determined a priori using the “*n:q* criterion” [36], a subject-per-parameter ratio criterion commonly applied in psychometric validation studies. In this study, *n* represents the number of participants, while *q* corresponds to the model parameters to be estimated (=40). A minimum of 400 participants was ensured to maintain a 10:1 subject-to-parameter ratio, which is considered an appropriate threshold for the main analysis of this study [36]. This ratio has been widely recommended in the psychometric literature as a balance between statistical power and model stability to enhance parameter estimation accuracy and minimize issues related to model overfitting or convergence problems. Given that our model includes 40 parameters, a sample size of at least 400 (10 subjects per 40 parameters; n_minimum_ = 400) participants aligns with these guidelines, ensuring robust and generalizable findings.

### 2.3. Procedure

The snowball sampling method was employed to gather participants from the general population through social media channels [37,38]. The inclusion criteria were established as follows: (A) participants must be at least 18 years old; (B) they should be native speakers of Italian; (C) they are required to provide complete answers; and (D) they must consent by signing an online informed consent form. This study was approved by the Ethics Committee of the Istituto Auxologico Italiano (protocol n° 03C020; Date of approval: 2020_02_18). All procedures complied with the ethical guidelines established by the institutional and/or national research committee and aligned with the principles of the 1964 Declaration of Helsinki and its subsequent amendments or equivalent ethical standards.

### 2.4. Participants

A total of 811 participants were enrolled in this study: 253 males (31.2%) and 558 females (68.8%) with ages ranging from 18 to 80 years (*mean* = 43.18, *SD* = 12.57). According to the inclusion/exclusion criteria, none of the questionnaires had missing values. A detailed description of the sample is reported in Table 1.

### 2.5. Measures

Socio-demographic information was gathered, covering age, level of education, and occupational status.

#### The New General Self-Efficacy Scale (NGSES)

The NGSES [2] is a self-report instrument specifically designed to measure one’s perceived general self-efficacy. The scale comprises eight items, each rated on a 5-point Likert scale ranging from 1 (=strongly disagree) to 5 (=strongly agree). The items were developed to assess the target construct regardless of the context, making the scale applicable to any type of investigation, whether in clinical, work, social, or everyday life settings. Examples of items include the following: *“I will be able to achieve most of the goals that I have set for myself”* and *“Even when things are tough, I can perform quite well”*. There are no reverse-scored items. To calculate the total score, simply sum the scores of all the items on the scale. The possible scores range from 8 (minimum) to 40 (maximum), representing the lowest to highest levels of perceived general self-efficacy. Higher scores indicate greater perceived self-efficacy.

### 2.6. Statistical Analysis

**Figure 1:**
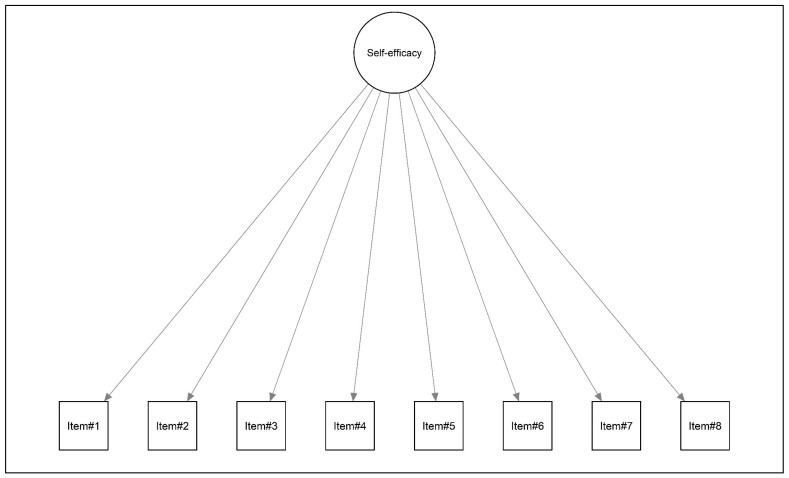
Conceptual representation of the NGSES.

The analysis was conducted using R software (v 4.3.2) [39] along with several packages, including lavaan [40], psych [41], semTools [42], and tidyverse [43]. To examine the factorial structure of the NGSES, a confirmatory factor analysis (CFA) was conducted. A single-factor model was specified based on prior validation studies [44], with each item loading onto a single general latent variable labeled ‘self-efficacy’ (see Figure 1).

Given the ordinal nature of the NGSES response format, the diagonally weighted least square (DWLS) estimator was applied for the CFA [36,44,45]. DWLS is specifically recommended for categorical or Likert-type data, as it provides more accurate parameter estimates and standard errors compared to maximum likelihood estimation, which assumes continuous and normally distributed data [36]. Model fit was assessed using several indices: the chi-square statistic (*χ*^2^), the Root Mean Square Error of Approximation (RMSEA), the Comparative Fit Index (CFI), and the Standardized Root Mean Residual (SRMR) [36,44,45]. The model’s goodness of fit was evaluated based on the following criteria: (A) a non-significance *χ*^2^ statistic, (B) an RMSEA value below 0.08, (C) a CFI greater than 0.95, and (D) an SRMR less than 0.08 [36,44,45,46].

Furthermore, the item discriminant power (IDP) was evaluated to assess the effectiveness of items in distinguishing between individuals exhibiting low and high levels of the construct being measured [47,48]. This process involved calculating the maximum possible total score for each scale and determining the quartile rank for each participant. Independent sample *t*-tests were then performed, along with effect size calculations (Cohen’s d), to evaluate item discrimination. This analysis utilized the total scale score as the dependent variable and categorized participants based on their lowest and highest quartiles [47,48]. Adjusted item-total correlations (r*_it-tot_*) were also computed [49], and internal consistency was assessed using McDonald’s omega [50] and maximal reliability (MR) [51,52,53,54].

Measurement invariance (MI) analysis was performed using the procedure recommended by Millsap and Tein (2004) to assess whether the factorial structure of the NGSES was consistent between males and. females [55,56,57]. Following the literature, the model structure was evaluated separately for each sample [58]. Once a satisfactory model fit was confirmed in both samples, four nested with increasing equality constraints were sequentially tested: Configural Invariance (M1: factorial structure was forced to equality across groups); Metric Invariance (M2: factorial structure and factor loadings were forced to equality across groups); Scalar Invariance (M3: factorial structure, item loadings, and intercepts were forced to equality across groups); and Latent Means Invariance (M4: factorial structure, item loadings and intercepts, and latent means were forced to equality across groups) [58,59]. These nested models were then compared to assess measurement invariance. Model comparisons were evaluated using the following: ΔCFI (<0.010) and ΔRMSEA (<0.015), which served as cutoff criteria for determining model equivalence [44,59,60]. Failure to meet these thresholds, along with a decline in model fit, was considered evidence of model inadequacy.

## 3. Results

### 3.1. Structural Validity

The NGSES demonstrated a good fit to the data. Although the *χ*^2^ statistic was statistically significant [*χ*^2^ (20) = 94.221; *p* < 0.001], all other fit indices indicated a well-fitting model: RMSEA = 0.068; 90% CI [0.054, 0.082], CFI = 0.994, SRMR = 0.043. As shown in Table 2, all item loadings were statistically significant, ranging from 0.597 (item#7) to 0.791 (item#5). The detailed results are presented in Table 2.

### 3.2. Psychometric Properties

**Figure 2:**
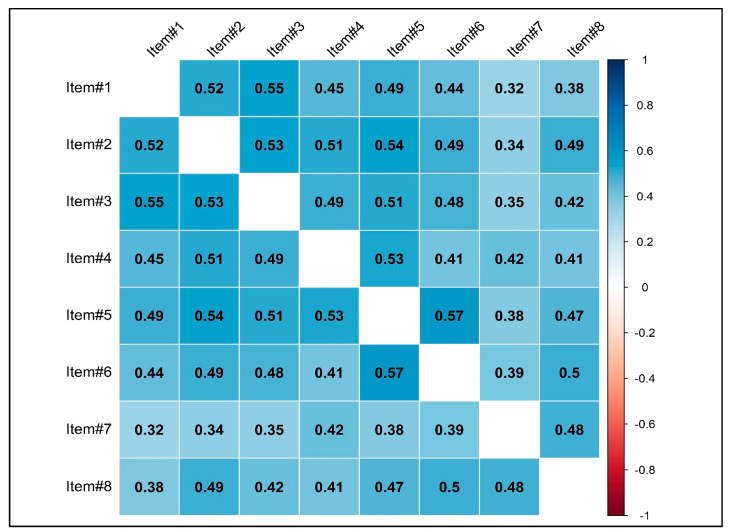
Correlation between NGSES items. *Note:* all correlations are statistically significant with *p* < 0.001.

As observed in the correlation matrix presented in Figure 2, the items of the NGSES are all significantly correlated with each other (*p* < 0.001) with none of them exceeding the critical threshold of 0.85. The IDP analysis indicated that all eight items effectively distinguished between individuals with a low or high level of the construct (Table 2). The discrimination parameter *t*_i_ ranged from |17.27| (item#7) to |27.09| (item#4), with corresponding Cohen’s *d* effect sizes between 1.71 and 2.69. Additionally, the adjusted item total correlation demonstrated a moderate-to-strong relationship between each item and the general ‘Self-efficacy’ factor. Regarding internal consistency, McDonald’s omega indicated strong reliability for the NGSES (ω = 0.872) as well as the MR coefficient (MR = 0.881).

### 3.3. Measurement Invariance

#### Gender (Male vs. Female)

*Model for males*: The *χ*^2^ statistic was statistically significant: *χ*^2^ (20) = 46.673; *p* < 0.001. All other fit indices indicated an acceptable model fit: RMSEA = 0.073, 90% CI [0.046, 0.100], CFI = 0.994, SRMR = 0.054.

*Model for females*: The *χ*^2^ statistic was statistically significant: *χ*^2^ (20) = 73.520; *p* < 0.001. All other fit indices suggested a good model fit: RMSEA = 0.069, 90% CI [0.053, 0.087], CFI = 0.994, and SRMR = 0.044.

*Configural Invariance*: The configural invariance model demonstrated good model fit indices, indicating that the factor structure was similar for males and females. While the *χ^2^* statistic was statistically significant [*χ*^2^ (40) = 120.194, *p* < 0.001], all other fit indices suggested a good fit to the data: RMSEA = 0.070, CFI = 0.994;, and SRMR = 0.047.

*Metric Invariance*: The metric invariance fit the data well. Although the *χ^2^* statistic remained statistically significant [*χ*^2^ (47) = 125.294, *p* < 0.001], the other fit indices confirmed a good fit: the RMSEA = 0.064, CFI = 0.994, and SRMR = 0.047. Additionally, the non-significant changes in the RMSEA (|ΔRMSEA| = 0.006) and CFI (|ΔCFI| = 0.000) indicated that the items loadings were equivalent across genders, suggesting that the items measured the latent factor consistently regardless of gender.

*Scalar Invariance*: The scalar invariance model also demonstrated good model fit indices. Despite the statistical significance of the χ^2^ statistic [*χ*^2^ (70) = 152.748, *p <* 0.001], the model fit remained strong: the RMSEA = 0.054, CFI = 0.994, and SRMR = 0.048. The non-significant decreases in RMSEA (|ΔRMSEA| = 0.010) and CFI (|ΔCFI| = 0.000) supported the assumption that males and females had equivalent item intercepts, meaning that individuals with the same level of the trait were expected to provide similar responses regardless of gender.

*Latent Means Invariance*: The latent mean invariance model showed a good fit to the data. Although the *χ*^2^ statistic was statistically significant [*χ*^2^ (71) = 153.683, *p* < 0.001], the fit indices showed a good model fit: the RMSEA = 0.054, CFI = 0.994, and SRMR = 0.048. Furthermore, the non-significant changes in RMSEA (|ΔRMSEA| = 0.000) and CFI (|ΔCFI| = 0.000) suggested that men and women had comparable expected latent means for the measured trait. Results are summarized in Table 3.

## 4. Discussion

This cross-sectional study aimed to test the structural validity and psychometric properties of the Italian version of the NGSES for the assessment of perceived GSE within a large general population sample.

CFA revealed a good fit to the data, supporting the robust psychometric properties of the one-factor structure. Furthermore, all items demonstrated statistically significant loadings, with values of *λ* ≥ 0.597. These findings are consistent with prior studies demonstrating a unidimensional structure on the NGSE with a good fit to empirical data [2,21,61].

Item correlations were moderately strong, suggesting good/high cohesion among the scale’s components without exceeding the critical threshold of 0.85 [49,62,63]. This suggests that, while all items measure self-efficacy, they reflect different facets of the construct, avoiding semantic redundancy.

Moreover, composite reliability, calculated using the omega coefficient [64], further confirmed the scale’s internal consistency (ω = 0.872) as well as the MR coefficient (MR = 0.881). These measures guarantee that the composite score derived from the eight items of the scale accurately and reliably assesses individuals’ perceived general self-efficacy.

This finding aligns with the internal consistency originally reported by Chen et al. (2001) [2] and is consistent with results from the Polish version of the NGSES [65], the Arabic version [66], and the newly developed Vietnamese version for individuals with colorectal cancer [67]. Furthermore, it surpasses the reliability levels reported for the Greek version [61], thereby reinforcing the scale’s reliability across diverse cultural contexts. Furthermore, it should be highlighted that, in the present study, only a single first-order factor structure was tested, and no alternative models were evaluated. The main reason for this choice lies in the fact that the semantic structure and content of the items do not support the possibility of a multifactorial structure, which could warrant the evaluation of alternative models. For this reason, no structures other than the first-order unidimensional model were tested.

Last, factorial invariance tests across genders were implemented. First, configural invariance was established, demonstrating good model fit indices. Thus, we may conclude that the broad conceptual meaning of the construct is the same in both males and females. Second, the achievement of metric invariance, obtained by constraining the equivalence of factor loadings, confirms that each item contributed similarly to the latent construct of self-efficacy across the two groups. The scalar invariance model, which tests whether males and females respond to the items equivalently at the same absolute level of the trait, also demonstrated a good fit to the data. Also, the latent means invariance model indicated that the latent mean of self-efficacy does not differ significantly between males and females. This means that, on average, both genders have similar levels of self-efficacy. This finding has important implications for the interpretation of self-efficacy scores, as it suggests that there is no bias in how self-efficacy is measured across gender groups.

Measurement invariance is a crucial requirement whether comparisons across groups are implemented in social sciences [68,69]. Indeed, as noted by Chan [1] (p. 108), «*We cannot assume the same construct is being assessed across groups by the same measure without tests of measurement invariance*».

This study provides robust evidence that the NGSES is a reliable and valid tool for assessing general self-efficacy across genders, allowing future research conducted in the Italian cultural context to properly examine differences in self-efficacy between males and females.

Findings from this study also highlight potential clinical implications following the NGSE adaptation. Tools like the NGSES can assess self-efficacy in both general and clinical populations, offering insights into its influence on health outcomes. For example, individuals with low self-efficacy may experience a negative cycle, where diminished confidence in managing health challenges leads to worsening physical and psychological conditions. Moreover, low perceived self-efficacy can hinder treatment adherence and access, further exacerbating health difficulties.

Nonetheless, this study has certain limitations. First, the reliance on social media platforms for recruitment may affect the generalizability of the findings. Also, another potential limitation concerns the choice of sample size, which may have led to an underestimation of the required number of participants. Although the method used to determine the minimum sample size for this study (i.e., the *n:q* rule) is widely supported in the literature [36], other approaches, such as simulation studies, might have provided more precise estimates of the required sample size. However, it is important to note that the present study is based on a sample size significantly larger than the one suggested by the initial rule. Consistently, despite the relatively large sample size, the sample is heterogeneous in terms of gender and educational level, with a notable prevalence of females. This imbalance may further limit the generalizability of the findings to broader populations, particularly in male-dominated or more educationally diverse groups. Future research should aim to recruit more balanced samples to ensure that the findings are representative and applicable across various demographic categories, thereby enhancing the external validity of the results. Additionally, the absence of longitudinal data prevented an assessment of test–retest reliability, and further research is needed to examine aspects such as convergent and discriminant validity.

Despite these limitations, this study has notable strengths. It represents the first attempt to assess the psychometric properties of the NGSES in the Italian general population, using rigorous and internationally endorsed statistical methods. The findings confirm the scale’s utility for both clinical and research purposes, offering a concise yet reliable alternative to lengthier self-efficacy measures.

## 5. Conclusions

The GSES was found to have a unidimensional structure and proved to be a valid and reliable instrument for measuring GSE within the Italian population. Furthermore, the scale exhibited strong psychometric properties for both males and females. As a result, the GSES can be effectively applied in both non-clinical and research settings; however, its effectiveness in clinical settings has yet to be evaluated. Indeed, its short format makes it a practical option in situations where time and resources are limited.

## Figures and Tables

**Table 1:** Sample descriptive statistics.

	**Descriptives**	
Age (*M*; *SD*)	43.07	12.572
Gender (*n*; %)		
Males	253	31.2%
Females	558	68.8%
Education (*n*; %)		
Middle school	100	12.3%
High school	384	47.3%
Bachelor degree	135	16.6%
Master degree	144	17.8%
Ph.D.	48	5.9%
Civil status (*n*; %)		
Single	159	19.6%
In a relationship	227	28.0%
Married	327	40.3%
Separated	31	3.8%
Divorced	52	6.4%
Widowed	15	1.8%
Work status (*n*; %)		
Full-time worker	404	49.8%
Part-time worker	116	14.3%
Entrepreneur	166	20.5%
Student	47	5.8%
Unemployed	49	6.0%
Retired	29	3.6%

**Table 2:** Item descriptive statistics, psychometric properties, and confirmatory factor analysis (CFA) results.

	Descriptive Statistics				Properties			CFA	
	***M***	***SD***	***SK***	***K***	***t***	***d***	***r*(it-tot)**	***λ***	***R* ^2^**
NGSES	29.044	4.740	−0.442	1.367					
Item#1	3.57	0.866	−0.532	0.289	−21.72	2.16	0.612	0.724	0.524
Item#2	3.69	0.822	−0.679	0.628	−22.30	2.22	0.676	0.788	0.621
Item#3	3.90	0.775	−0.826	1.254	−19.64	1.97	0.654	0.775	0.600
Item#4	3.33	0.934	−0.198	−0.378	−27.09	2.69	0.631	0.725	0.526
Item#5	3.64	0.827	−0.473	0.415	−23.08	2.30	0.688	0.791	0.625
Item#6	3.89	0.743	−0.909	1.714	−17.99	1.80	0.637	0.760	0.577
Item#7	3.31	0.836	−0.101	0.008	−17.27	1.71	0.510	0.597	0.356
Item#8	3.71	0.725	−0.675	0.952	−18.55	1.85	0.611	0.721	0.520

*Note: M* = Mean; *SD* = standard deviation; *SK* = skewness; *K* = Kurtosis; *t* = *t*-test; *d* = Cohen’s *d*; *r*(it-tot) = adjusted item-total correlation; *λ* = standardized factor loading; and *R*^2^ = item explained variance.

**Table 3:** Measurement invariance across genders.

	*χ*^2^ (*df*)	RMSEA	CFI	SRMR	ΔRMSEA	ΔCFI	ΔSRMR
Model males							
Model females							
Configural inv.	120.194 (40)	0.070	0.994	0.047			
Metric inv.	125.294 (47)	0.064	0.994	0.047	−0.006	0.000	0.000
Scalar inv.	152.748 (70)	0.054	0.994	0.048	−0.010	0.000	0.001
Latent means inv.	153.683 (71)	0.054	0.994	0.048	0.000	0.000	0.000

*Note:* Model ‘males’ *n* = 253; Model ‘female’ *n* = 558; *χ*^2^ = chi-square test; *df* = degrees of freedom; RMSEA = Root Mean Square Error Of Approximation; CFI = Comparative Fit Index; SRMR = Standardized Root Mean Residual; and Δ(…) = differences between indices.

## Data Availability

Data are available upon reasonable request due to privacy and ethical restrictions.

## Supplementary Materials

The following supporting information can be downloaded at https://www.mdpi.com/article/10.3390/jcm14061988/s1.
