# Supplementary material for: The Italian Version of the New General Self-Efficacy Scale (NGSES): Structural Validity, Psychometric Properties, and Measurement Invariance
Source: J Clin Med. 2025 Mar 14;14(6):1988. doi: 10.3390/jcm14061988 (PMC11943235; doi:10.3390/jcm14061988)
Supplement: Supplementary file 1 [file jcm-14-01988-s001.zip › jcm-3485373-supplementary.pdf]

NEW GENERAL SELF-EFFICACY SCALE (GSE)

ISTRUZIONI: Pensando a se stesso/a, indichi quanto è d'accordo con le affermazioni che seguono.

ATTENZIONE: la preghiamo di provare a rispondere a tutte le domande nel modo più preciso possibile

1	2	3	4	5
Fortemente in disaccordo	In disaccordo	Né in accordo né in disaccordo	In accordo	Fortemente in accordo

1	Sarò in grado di raggiungere la maggior parte degli obiettivi che mi sono prefissato/a	1	2	3	4	5
2	Di fronte a compiti difficili, sono certo/a che li porterò a termine	1	2	3	4	5
3	In generale, penso di poter ottenere risultati che siano importanti per me	1	2	3	4	5
4	Credo di poter ottenere il massimo in ogni compito che mi prefisso di realizzare	1	2	3	4	5
5	Sarò in grado di superare con successo molte sfide	1	2	3	4	5
6	Sono fiducioso/a di poter svolgere in maniera efficace molti compiti diversi	1	2	3	4	5
7	In confronto agli altri, sono in grado di svolgere bene la maggior parte dei compiti	1	2	3	4	5
8	Anche quando le cose sono difficili, sono in grado di svolgerle abbastanza bene	1	2	3	4	5
